# Circulating Tumor Cells in Genitourinary Malignancies: An Evolving Path to Precision Medicine

**DOI:** 10.3389/fonc.2017.00006

**Published:** 2017-01-27

**Authors:** Cory M. Hugen, Daniel E. Zainfeld, Amir Goldkorn

**Affiliations:** ^1^Keck School of Medicine and Norris Comprehensive Cancer Center, Urology, Los Angeles, CA, USA; ^2^Keck School of Medicine and Norris Comprehensive Cancer Center, Medicine, Los Angeles, CA, USA

**Keywords:** circulating tumor cells, bladder cancer, prostate cancer, liquid biopsy, molecular characterization

## Abstract

Precision medicine with molecularly directed therapeutics is rapidly expanding in all subspecialties of oncology. Molecular analysis and treatment monitoring require tumor tissue, but resections or biopsies are not always feasible due to tumor location, patient safety, and cost. Circulating tumor cells (CTCs) offer a safe, low-cost, and repeatable tissue source as an alternative to invasive biopsies. “Liquid biopsies” can be collected from a peripheral blood draw and analyzed to isolate, enumerate, and molecularly characterize CTCs. While there is deserved excitement surrounding new CTC technologies, studies are ongoing to determine whether these cells can provide reliable and accurate information about molecular drivers of cancer progression and inform treatment decisions. This review focuses on the current status of CTCs in genitourinary (GU) cancer. We will review currently used methodologies to isolate and detect CTCs, their use as predictive biomarkers, and highlight emerging research and applications of CTC analysis in GU malignancies.

## Introduction

Circulating tumor cells (CTCs) are cancer cells that are shed from a primary or metastatic tumor site, traffic through the vasculature, and may establish distant metastasis. CTCs were first reported as far back as the late 1800s: Ashworth detected the cells in blood drawn from the saphenous vein and postulated that they would be identical to the primary tumor (1). However, CTCs began to make a significant scientific and clinical impact on cancer only in the recent decade, enabled by technological advancements that allow improved detection and isolation of these rare cells from blood.

Circulating tumor cells have been identified and isolated from patients with virtually every type of solid malignancy. Cancers of the prostate, bladder, and kidney comprise 3 of 10 most common primary malignancies diagnosed in the United States (2), and CTCs have been isolated from each of these malignancies. While numerous studies have focused on CTCs in patients with prostate cancer, few studies have focused on other genitourinary (GU) malignancies including bladder, kidney, and testicular.

In this review, we will focus on CTCs with respect to their role in GU malignancies. We will first provide a brief synopsis highlighting some of the currently available technologies used to identify and recover CTCs. We will then review how CTCs are being developed clinically as prognostic and predictive biomarkers in GU malignancies. Finally, we will address how CTCs are being leveraged to elucidate disease biology by identifying key mechanisms of resistance and progression.

## Brief Synopsis of Technologies

**Figure 1:**
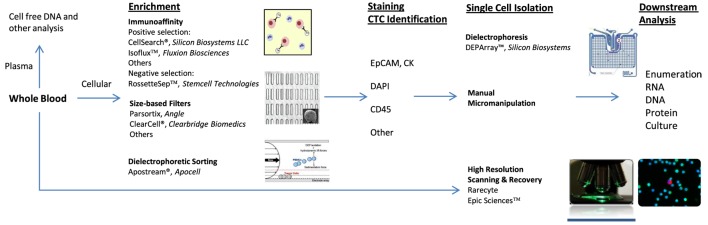
**Circulating tumor cell (CTC) identification and isolation**. CTC workflows: immunoaffinity generally utilizes antibody to epithelial cell adhesion molecule and is dependent on EPCAM+ CTCs. Size-based filters are reliant on typically larger size of CTCs. All techniques require some form of staining for cellular identification. Common stains include cytokeratin, 4′,6-diamidino-2-phenylindole (DAPI), leukocyte common antigen (CD45), and others. DEPArray™ enables isolation of individual, intact cells in dielectric cages. Apostream^®^ allows for antibody-independent capture of CTCs. Epic Sciences™ places unenriched sample on proprietary slide prior to CTC staining and high-resolution scanning. Downstream analysis opportunities are numerous and continue to be explored.

Several different methods have been developed to isolate and analyze CTCs. Each detection strategy exploits a different physical property of the cells in order to separate them from the billions of red blood cells and millions of white blood cells (WBC) also present in a standard 7.5 ml sample of human blood (method overview in Figure 1).

The most clinically established method to isolate CTCs is immunoaffinity (3). This method employs magnetic beads attached to antibodies directed at specific cell surface antigens present on CTCs, most commonly epithelial cell adhesion molecule (EpCAM). After the antibodies bind to their target, a magnet is used to separate these cells from background cells. The most heavily studied immunoaffinity device has been the CellSearch^®^ platform (developed by Janssen Diagnostics, LLC and recently acquired by Menarini-Silicon Biosytems). This FDA-cleared system uses ferrofluid nanoparticles attached to antibodies directed against EpCAM on CTCs to separate these cells from other cells present in the buffy coat following centrifugation of whole blood. Once separated, these cells are then stained with fluorophore-labeled antibodies directed against cytokeratin (CK), CD45 (a leukocyte-specific cell marker), and the 4′,6-diamidino-2-phenylindole (DAPI) nuclear stain. Cells that stain EpCAM+, CK+, CD45−, with DAPI+ are considered CTCs (4).

Another example of a system-utilizing immunoaffinity is the CTC-chip platform. The CTC-chip has thousands of small posts embedded with EpCAM antibodies. Blood flows through the chip and cells expressing EpCAM on their surface bind to the posts and are separated from non-EpCAM expressing cells (5, 6). The CTC-iChip uses immunoaffinity combined with microfluidics to isolate CTCs but does not rely on EpCAM labeling. This device is composed of two chips that first separate nucleated cells present in whole blood from non-nucleated cells using deterministic lateral displacement which separates cells *via* size-based deflection. The WBCs are also tagged with CD45 and CD66b (a more specific target of granulocytes) antibodies during this phase. The second step uses inertial focusing and magnetophoresis to separate the remaining nucleated cells based on their magnetic bead load, which separates CTCs from WBCs (7).

Immunoaffinity-based techniques have advantages as well as limitations: the most notable strength of these approaches is their extensive validation to date. CellSearch has by far been the most commonly used method in large clinical trials across malignancies, where it has been validated repeatedly and become a *de facto* “gold standard” to which emerging technologies are compared. If a tumor-specific cell surface marker is available for a given malignancy, immunoaffinity can serve as a sensitive and specific strategy for enriching cancer cells from the blood. Conversely, the main limitation of immunoaffinity-based techniques is that such a sensitive and specific marker often is not available. EpCAM-based systems may fail to identify subsets of cells undergoing epithelial-to-mesenchymal transition (EMT). This phenotypic transformation is thought to be necessary for cells to migrate from the primary tumor location and metastasize. These cells often downregulate EpCAM antigen and express mesenchymal antigens instead, thus reducing the sensitivity of an EpCAM-based approach. Moreover, EpCAM is not a CTC-specific marker *per se*, but rather a marker of all epithelial cells; thus, it is possible that some CTCs enriched in this manner may not be tumor cells but rather benign epithelial cells, thus reducing the specificity of EpCAM-based enrichment.

A second broad methodology used to isolate CTCs is based on size and deformability, as CTCs are typically larger and more rigid than WBCs. Many groups have described variations of this technique, and some examples include use of a Parylene-C slot microfilter (8), a porous polycarbonate membrane (9), a resettable trap with adjustable aperture (10), and a polycarbonate track-etch-type membrane with cylindrical pores (11).

Size-based techniques offer several pros and cons: one advantage compared with immunoaffinity-based technologies is that cell isolation does not rely on cell surface markers, allowing for the capture of cells that do not express the platform-specific antigen. Another advantage, compared with CellSearch^®^, is that cells do not require fixation and therefore live cells can be captured and further manipulated. On the other hand, size-based platforms have limited sensitivity and specificity, because they may retain large cells that are not CTCs or fail to capture smaller CTCs. Additionally, once these cells are captured, they require additional positive identification steps (e.g., immunofluorescent staining) (3).

Several other methodologies exist to capture and identify CTCs based on a variety of cellular features. Examples include the DEPArray™ (Silicon Biosystems, Italy) system, which uses a microfluidic cartridge with controllable electrodes to create dielectric cages around cells for isolation and recovery (12). Another dielectric separation-based system is ApoStream^®^ (Apocell Inc., Houston, TX, USA), which also exploits the differences in dielectric properties between CTCs and peripheral blood mononuclear cells to focus and separate cells (13). Others have isolated CTCs based on their ability to invade collagenous matrices, or using atomic force microscopy to determine the nanomechanical properties of CTCs such as elasticity, deformation, and adhesion to identify CTCs (14, 15). All of these approaches offer unique advantages and disadvantages based on sensitivity, specificity, speed, and reproducibility of workflow, and compatibility with fixed vs live cells. In-depth discussion of each is beyond the scope of this review.

More recently, two platforms have eschewed enrichment altogether and instead take a “no cell left behind” approach using rapid high-resolution scanning and automated detection algorithms to identify CTCs. Epic™ (Epic Sciences, San Diego, CA, USA) utilizes immunofluorescent staining (CK, DAPI, CD45, as well as one or two additional antibodies) of nucleated cells spread as a monolayer on proprietary slides. These slides are then scanned using a whole slide fluorescent microscope. A computerized algorithm then incorporates immunofluorescent and morphologic features to identify candidate CTCs (16). Rarecyte™ (RareCyte, Seattle, WA, USA) spreads the buffy coat on slides and then performs automated multiplex imaging with scanning algorithms to detect and rank potential CTCs (17). Advantages of these systems include rapid detection, automation, and the ability to map and characterize the entire complement of circulating cells, regardless of size or antigen expression. However, the cells are fixed to a solid matrix that somewhat limits manipulation, precludes live cell assays, and allows recovery of most but not all DNA and RNA for analysis.

## Clinical Development

### Prostate Cancer

The majority of CTC research and clinical development to date in GU malignancies has been undertaken in patients with prostate cancer, and most of these studies have used CTC enumeration as their clinical end point. Beyond enumeration, recent studies have begun to molecularly characterize isolated CTCs and to study their nanomechanical properties as enabling downstream technologies continue to develop. Prostate cancer CTC enumeration studies have been performed in the full spectrum of disease states, from localized prostate cancer (18, 19), to metastatic hormone-sensitive prostate cancer (mHSPC) (20–22), to metastatic castrate-resistant prostate cancer (mCRPC) (23–28). In the following sections, we will review studies in each of these areas (selected prospective studies highlighted in Table 1).

**Table 1:** **Selected prospective circulating tumor cell (CTC) studies in prostate cancer**.

Localized prostate cancer	
Davis et al. (18)	97 patients with localized prostate cancer evaluated for CTCs prior to planned prostatectomy as well as various intervals following surgery. CTCs detected in 21% of patients and did not correlate with tumar volume, pathological stage, or Gleason score
Pal et al. (19)	Sample blood from 35 patients with high-risk localized prostate cancer collected prior to treatment. CTCs identified in 49% prior to surgery but no correlation between CTC count and biochemical recurrence identified at 1 year follow-up
**Metastatic hormone-sensitive prostate cancer (mHSPC)**	
Okegawa et al. (21)	SO patients identified prior to initiation of hormonal therapy. CTC counts ranged from 0 to 222/7.5 ml blood, with 44 patients having 5 or more CTCs. More than 5 CTCs median androgen deprivation therapy (ADT) responsiveness 17 months vs 32 for those with <5CTCs
Goldkorn et al. (35)	CTCs detectable in 78/211 (37%) patients in CTC enumeration corollary to SWOG 1216 (ADT plus bicalutamide or orteronel in setting of MHSPC). Baseline CTC detection associated with higher prostate-specific antigen (PSA), extensive disease, and bony metastasis. Trial ongoing including molecular characterization
**Metastatic castrate-resistant prostate cancer (mCRPC)**	
de Bono et al. (24)	Evaluation of 231 patients prior to initiating new line chemotherapy and monthly thereafter. Patients stratified to favorable (<5) vs unfavorable (>5) CTC counts. CTC count superior to PSA for predicting OS (11.5 vs 21.7 months) among those with unfavorable CTC count
Scher et al. (25)	147 eligible patients from IMMC38 trial identified prior to initiation of chemotherapy. Changes in CTC number strongly associated with risk of death at 4, 8, and 12 weeks after treatment suggesting CTC number as a continuous variable useful for disease status monitoring
Goldkorn et al. (29, 30)	Prognostic value of CTCs for overall survival and disease response assessed in SWOG 0421(docetaxel plus prednisone with or without atrasentan). Baseline CTC counts found to be prognostic of overall survival and rising CTCs at 3 weeks predicted worse OS. In addition, telomerase activity in CTCs captured on Parylene-C microfilter found prognostic of overall survival (19 vs 12 months) in men with 6–54 CTCs/7.5 ml
Antonarakis et al. (39)	RT-PCR identified androgen receptor splice variant 7 (AR-V7) splice variant in CTCs of patients receiving enzalutamide or abiraterone for mCRPC. AR-V7 in CTCs associated with lower PSA response, shorter PFS, and decreased OS

#### Castrate-Resistant Prostate Cancer

Therapeutic options for mCRPC have expanded markedly over the past decade and now include several new hormonal therapies, chemotherapies, a vaccine, and a radiopharmaceutical. Informative new biomarkers may help guide choice of therapy. In one of the first published studies, Danila et al. (25) enumerated CTCs from 120 patients with mCRPC using the CellSearch^®^ platform and showed that CTC enumeration, when treated as a continuous variable, was inversely associated with overall survival. The combination of CTC enumeration, prostate-specific antigen (PSA), and albumin was found to be a better predictor of survival than any of these individual variables alone. CTC counts were higher in patients with bone vs soft-tissue metastasis and in those who received prior cytotoxic chemotherapy.

In a seminal mCRPC study by de Bono et al. (24), CellSearch^®^ was used to enumerate CTCs in 276 patients with metastatic CRPC treated with various hormonal agents and chemotherapies. CTCs were detected in 231 patients, and patients with ≥5 CTCs had significantly worse overall survival than patients with <5 CTCs (11.5 vs 21.7 months, HR 3.3, *p* < 0.0001). This CTC cutoff value was also a better predictor of overall survival than PSA at 1 year. Interestingly, patients whose CTCs increased from <5 at baseline to ≥5 at subsequent time points had worse outcomes, whereas patients who converted from ≥5 to <5 CTCs had better outcomes than patients who remained stable. Goldkorn et al. (29) confirmed this CTC cut point in a prospective randomized phase III study, SWOG 0421. Patients with mCRPC were treated with docetaxel ± atrasentan, and those with baseline CTC counts of ≥5 vs <5 had median overall survival differences of 13 vs 26 months, respectively (HR, 2.74, 95% CI, 1.72–4.37, *p* < 0.001). Moreover, any increase in the CTC count after cycle 1 of therapy (at 3 weeks from baseline) was associated with reduced overall survival (HR, 2.55, 95% CI, 1.04–6.24, *p* = 0.041). In a parallel study, Goldkorn et al. (30) evaluated telomerase activity in CTCs isolated from patients enrolled in SWOG 0421 and found that this cancer marker predicted overall survival in patients with CTC counts ≥5. While de Bono and Goldkorn arrived at a cutoff of ≥5 or <5 CTCs, Goodman et al. (23) performed threshold analysis from 100 patients with mCRPC and found that a CTC count of 4 was the optimal cutoff that best correlated with overall survival.

In a reanalysis of the 231-patient IMMC38 cohort described earlier (24), Scher et al. (26) found that dynamic CTC enumeration monitoring at predefined “landmark” time points could be used to monitor disease status. CTC counts of ≥5 vs <5 at 4, 8, and 12 weeks following initiation of chemotherapy were associated with risk of death from prostate cancer, and CTC counts analyzed as a continuous variable (rather than specific cut points) also correlated with outcome.

A separate study by the same group (31) specifically evaluated CTC enumeration as a surrogate for overall survival. This was a secondary objective of COU-AA-301, a multinational, randomized double-blind phase III trial of abiraterone acetate plus prednisone vs prednisone alone in patients with metastatic CRPC who were previously treated with docetaxel. CTC enumeration combined with lactate dehydrogenase values met the Prentice criteria for surrogacy in a predictive marker. Such fully validated surrogate biomarkers have the potential to shorten drug development times and potentially guide treatment choices, thus minimizing morbidity from non-efficacious treatment at an earlier time point and maximizing the potential therapeutic efficacy from another agent.

#### Hormone-Sensitive Prostate Cancer

Circulating tumor cell analysis may prove useful in mHSPC as well, especially given recent studies demonstrating the benefit of combining hormonal therapy with chemotherapy (32, 33). Yu et al. (34) performed a corollary study as part of SWOG S0925, a randomized phase II trial of androgen deprivation therapy (ADT) combined with the IGF1 antibody cixutumumab vs ADT alone in patients with metastatic HSPC. They showed that baseline CTC enumeration categories of 0, 1–4, and ≥5 for 39 evaluable patients was associated with PSA categories of ≤0.2, >0.2 to ≤4.0, and >4.0 at 28 weeks, *p* = 0.036. Of note, 16 (41%) patients had undetectable CTC values at baseline. Goodman et al. (20) evaluated multiple clinical variables including CTC enumeration in 33 patients with mHSPC and demonstrated that only baseline CTC count was independently prognostic of progression to mCRPC. They found that ≥3 vs <3 was the optimal enumeration cutoff and this predicted a shortened time to mCRPC.

Another study (21) demonstrated that a baseline cutoff of 5 CTCs in a cohort of 80 patients with mHSPC was an independent predictor of duration of ADT responsiveness (17 vs 32 months, *p* = 0.007). Furthermore, baseline PSA value was not a predictor of responsiveness; however, nadir PSA was a predictor. Additionally, a change in CTC count while on ADT was associated with treatment response duration: response-free rates for patients with CTC counts ≥5 vs <5 at all time points was 32 vs 12 months (*p* < 0.001) and for patients with ≥5 baseline CTC count who decreased to <5 during treatment it was 26 vs 12 months (*p* = 0.038). These results suggest that with additional validation, dynamic monitoring of CTC counts in HSPC may potentially be used as a clinical end point to guide changes in treatment strategy at an earlier point, possibly with improved outcomes.

Preliminary results of CTC studies in SWOG S1216, a randomized prospective phase III trial of MHSPC patients randomized to ADT in combination with either bicalutamide or orteronel, were recently presented at the 2016 American Society of Clinical Oncology meeting. Goldkorn et al. (35) demonstrated that CTCs were detectable in 78 of 211 (37%) of evaluable samples, and detection rates were impacted by whether patients were treatment naïve (41% detection) or had already initiated hormonal therapy (29% detection) at time of baseline draw, *p* = 0.01. Additionally, baseline CTC detection was associated with higher PSA (*p* = 0.03), presence of extensive disease (*p* < 0.001), and bony metastasis (*p* = 0.05). Collectively, these enumeration studies are establishing an association between baseline CTC counts and other baseline prognostic factors, as well as with time to progression to mCRPC.

#### Localized Disease

The majority of studies have focused on metastatic disease based on the expectation of finding more CTCs with greater disease burden. However, several studies have evaluated CTCs in the context of localized prostate cancer with the goal of predicting recurrence after local definitive therapy. Pal et al. (19) collected blood from 35 patients with high-risk localized prostate cancer and evaluated using CellSearch^®^ to discover whether CTCs from this group expressed any mesenchymal cell markers that could indicate that these cells were undergoing EMT. Blood was collected 2 weeks prior to radical prostatectomy, at the time of surgery, and 1 and 3 months following surgery and enriched CTCs were stained for the mesenchymal markers CD133 and E-cadherin. CTCs were detected in 49% of the 35 enrolled patients prior to surgery, and the percentages of CD133 and E-cadherin-positive CTC fragments were significantly higher in patients with biochemical recurrence at 1 year (*p* = 0.028 and 0.006, respectively). This detection rate is similar to the 52% detection rate noted by Kolostova et al. (9) in clinically localized prostate cancer.

Davis et al. (18) used CellSearch^®^ preoperatively in 97 patients with clinically localized prostate cancer and detected CTCs in 21% of patients. Interestingly, they also detected CTCs in 20% of the control group who had a negative extended-core prostate biopsy indicative of the inherent difficulties in CTC identification, particularly when greater volumes of blood (30 ml in this study) are utilized increasing the potential inclusion of benign epithelial cells in the blood of healthy individuals. The authors suggest that CTCs might be used to determine which patients should undergo a repeat prostate biopsy following negative initial biopsy, an approach that warrants further investigation.

In the only systematic review and meta-analysis we identified in this field, Ma et al. (36) concluded that CTC counts are associated with overall survival and disease-free survival in patients with localized prostate cancer.

### Molecular Characterization

As the sociologist William Bruce Cameron wrote: “not everything that can be counted counts, and not everything that counts can be counted”—CTC enumeration studies have provided a proof of concept for the detection and isolation of CTCs in humans, but the field is quickly moving beyond enumeration alone, with major efforts now focused on the molecular characterization of CTCs. Such studies aim to identify molecular drivers of treatment resistance or disease progression that can be used to monitor disease in real time and to direct personalized therapeutics.

#### Immunofluorescent Staining

As described earlier, the majority of CTC studies to date have employed immunofluorescence to some degree, whether simply to identify CTCs (EpCAM, CK, DAPI, CD45) or to characterize them (CD133, E-cadherin). Other examples include fluorescence *in situ* hybridation (FISH) (37) to detect *ERG* rearrangements, *PTEN* loss, and *AR* copy numbers (38). Antonarakis et al. (39) investigated androgen receptor splice variant 7 (AR-V7) in CTCs. The Alere™ CTC AdnaTest (Alere Inc., San Diego, CA, USA) was used to enrich CTCs for real-time PCR detection of AR-V7 and full-length androgen receptor (AR). Presence of AR-V7 in CTCs was associated with resistance to abiraterone and enzalutamide. This work was confirmed and extended recently by Scher et al. who obtained blood samples from men with mCRPC undergoing a change in systemic therapy due to progressive disease and identified CTCs using the EPIC Sciences platform. AR-V7 presence detected by immunofluorescent staining was predictive of poor response to AR signaling inhibitors and improved response to taxane therapy, suggesting a potential role for CTC evaluation in directing advanced prostate cancer therapy (40). At least four separate groups have evaluated AR expression in CTCs. Miyamoto et al. (41) showed that patients with metastatic prostate cancer initiating ADT had significantly different AR staining patterns compared with metastatic CRPC patients initiating second-line therapies for CRPC. Reyes et al. used fluorescence-activated cell sorting (FACS) and ImageStreamX to produce real-time high-resolution images of cells and demonstrated that AR expression was increased in patients with prior exposure to abiraterone and associated with increased Ki-67, a known cellular proliferation marker (42). On the other hand, Crespo et al. isolated CTCs using CellSearch^®^ and then used FISH to demonstrate that AR expression in CTCs was unchanged in patients treated with enzalutamide or abiraterone, confirming these findings in tissue samples (43). Finally, Darshan et al. demonstrated *via* immunofluorescence that AR localization in the CTC cytoplasm vs nucleus was associated with treatment response to chemotherapy (44). Therefore, monitoring CTC AR subcellular localization might be a useful clinical parameter for patients being treated with taxane based chemotherapy.

#### DNA/RNA Analysis

Increasingly, targeted and high-throughput methods are applied to amplified genetic material from rare cells captured from blood. Stott et al. (6) used CTC-chip followed by on-chip lysis to isolate RNA for RT-PCR evaluation of *TMPRSS2-ERG* translocation in metastatic patients to further the molecular characterization of prostate cancer CTCs. Punnoose et al. (45) used the Epic Sciences platform to identify CTCs and found that *PTEN* status in CTCs correlated with *PTEN* status in patient-matched CRPC tissue and that loss of *PTEN* in CTCs was associated with worse clinical outcomes. Miyamoto et al. (46) used CTC-iChip to isolate 77 CTCs from patients with prostate cancer. These cells were micromanipulated, and the RNA content was extracted for amplification and next-generation sequencing. Considerable heterogeneity existed between individual CTCs with regard to expression of AR mutations and splicing variants—even between cells isolated from the same patient. Non-canonical Wnt5a was found to play an important role in overcoming the antiproliferative effect of AR inhibition. Jiang et al. (47) performed whole-genome sequencing on CTCs of patients with advanced prostate cancer by combining the NanoVelcro CTC-chip with laser capture microdissection to identify shared genomic alterations between CTCs and tumor tissues including structural variants in PTEN, RB1, and BRCA2. Similarly, Lohr et al. (48) performed whole-exome sequencing on CTCs isolated from patients with metastatic prostate cancer as well as matched metastatic and primary tumor. 51/73 (70%) of CTC mutations were observed in matched tissue. In addition, 90 and 73% of 10 early-trunk and 56 metastatic-trunk mutations, respectively, found in the non-CTC tumor sample were also identified in CTC exomes.

An ongoing study embedded in a phase III prospective clinical trial (SWOG 1216) employs multiparametric CTC profiling to illuminate mechanisms of androgen therapy resistance over time. CTCs are not only being enumerated but also enriched for targeted sequencing (Liquid Biopsy platform, Cynvenio) as well as recovered for gene expression analysis (DEPArray platform, Silicon Biosystems). Studies such as this will ultimately contribute to more informed and effective therapy selection in the mHSPC disease state.

#### CTC Culture

Expansion of CTCs *in vitro* or in mouse avatar models holds potential for yielding large numbers of sustainable cells for molecular analysis and functional assays (e.g., drug sensitivity). This approach has met with some success in small cell lung cancer (49) and breast cancer (50). Unfortunately, experience has been more limited in prostate cancer, perhaps due to underlying differences in the biologies of these tumors, or due to lower abundance of recoverable CTCs. In one notable success, Gao et al. (51) were able to generate an organoid from CTCs isolated from a patient with metastatic prostate cancer. Whole-exome DNA sequencing demonstrated that this organoid expressed many of the same mutations present in archived formalin-fixed paraffin-embedded lymph node metastasis, but also with a few additional mutations. The authors suggest that these additional mutations may have been acquired during the transition from hormone-sensitive to castrate-resistant disease. Likewise, Vidal et al. successfully established CTC-derived xenografts in order to examine the role of GATA2 in regulating prostate cancer progression and chemotherapy resistance. CD45-negative cells were isolated from the blood of CRPC patients after separation by Ficoll gradient using FACS and subsequently implanted into NSG mice demonstrating potential utilities of these techniques (52).

Another group used the MetaCell^®^ system (MetaCell s.r.o., Ostrava, Czech Republic) to isolate viable intact CTCs. This system uses a porous polycarbonate membrane to isolate cells based on size, successfully detecting CTCs at a high rate, 25/39 (64%) of patients with muscle-invasive bladder cancer. They then successfully separated and cultured those cells *in vitro* in patients with prostate (9) and bladder cancer (53). Despite these encouraging findings, culturing CTCs from patients with GU cancers continues to be highly technically challenging, and multiple groups continue to work to develop improved methods. Ultimately, validation of such methods would require reliable culture techniques that could offer clinical utility for a significant portion of patients, and confidence that cultured CTCs closely represent the underlying molecular phenotype of patients’ disease.

### Bladder Cancer

To date, the majority of GU CTC research has been conducted in prostate cancer, probably due to its prevalence, the availability of large trial cohorts, and the presence of known molecular markers. All of these are present to a lesser degree in bladder cancer; nevertheless, as technologies improve and new treatments (e.g., immune checkpoint inhibitors) become available in bladder cancer, growing efforts are now invested in liquid biopsy and CTC studies in this disease.

#### Metastatic Disease

Gallagher et al. (54) used CellSearch^®^ to enrich CTCs from 33 patients with metastatic urothelial cell carcinoma (UCC) and detected CTCs in 14/33 (44%) of patients. Median CTC count in patients with two or more sites of metastasis was 3.5 vs 0 in patients with one metastatic site (*p* = 0.04). Okegawa et al. (55) used CellSearch^®^ to enumerate CTCs in patients with bladder cancer. CTCs were detected in 11/20 (55%) patients with metastatic bladder cancer and in 0/16 patients with non-metastatic UCC. Similarly, Naoe and colleagues (56) reported a 57% (8/14) detection rate in patients with metastatic disease. While detection rates do appear to correlate generally with disease state, the true CTC positivity may be impacted by variable EpCAM expression that may limit detection in these immunoaffinity-based studies. Flaig et al. (57) used the CellSearch^®^ platform and detected ≥1 CTC in 5/30 of patients with localized and 7/14 of patients with metastatic UCC. They also performed FISH using the UroVysion (Abbott Molecular, Des Plaines, IL, USA) probe set on 18 unique samples collected from the CellSearch^®^ cartridge looking for DNA copy number variation (CNV). Nine of these samples were collected from patients with detectable CTCs and CNV was detected in five samples. None of the nine samples collected from patients without CTCs had CNV. All patients with metastatic disease and detectable CTCs were dead at <1 year while only 3/7 patients with metastases but without detectable CTCs were deceased at <1 year.

While detection rates do appear to correlate generally with disease state, the true CTC positivity may be impacted by variable EpCAM expression that may limit detection in these immunoaffinity-based studies. Using the Epic Sciences platform including immunofluorescent staining and algorithmic scanning, Anantharaman et al. (58) examined blood samples from patients with metastatic bladder cancer and detected CTCs in 20/25 (80%) patients. CK^−^ CTCs were present in 14/25 (56%). Programed death-ligand 1 expression was found in both CK^+^ and CK^−^ cells and was associated with decreased overall survival. This study illustrates how high content imaging without enrichment may identify and characterize additional CTC subpopulations and help to guide therapy.

#### Localized Disease

Gazzaniga et al. (59) collected blood samples from 102 patients with T1G3 UCC prior to undergoing transurethral resection of bladder tumor. Using the CellSearch^®^ platform, CTCs were detected in 20% of patients and their presence was associated with time-to-first recurrence, progression-free survival, and the development of distant metastasis. In an earlier report from this same group on 44 patients with non-muscle-invasive bladder cancer, CTCs were detected in 8/44 (18%) of patients, and their presence was associated with higher tumor stage and the presence of carcinoma *in situ* (60).

Circulating tumor cell enumeration has also been studied in patients undergoing radical cystectomy. Guzzo et al. (61) detected CTCs in 9/43 (21%) of patients undergoing radical cystectomy for clinically localized disease. The presence of detectable CTCs was not associated with extravesical tumor staging on final pathology. Rink et al. (62) collected blood from 100 patients with non-metastatic UCC treated with radical cystectomy. They detected CTCs using CellSearch^®^ in 23 patients and reported that patients with detectable CTCs had worse recurrence-free survival, cancer-specific survival, and overall survival. They also stained for HER2 expression in the primary tumor, CTCs, and lymph nodes. Of the 22 CTC positive samples evaluated, HER2 expression was concordant with the primary tumor in 14 (64%) cases. For five cases with detectable CTCs and metastatic lymph nodes, there was 100% HER2 concordance rate (all stained negative).

Alva et al. (63) used the Isoflux™ immunomagnetic enrichment platform to detect CTCs in patients with UCC and compared their results to CellSearch^®^, finding improved CTC identification capacity. >10 CTCs was predictive of unfavorable pathology (pT1 or greater) from radical cystectomy following neoadjuvant chemotherapy (89% PPV) while CTC enumeration <10 was predictive of favorable pathology (57% NPV). In addition, they were able to detect somatic variants from 4/8 samples using next-generation sequencing from spike in samples. Sensitivity and specificity in the identification of CTCs using this platform will ultimately require further evaluation in the context of larger prospective cohorts. However, the detection of single-nucleotide variants from enriched cells provide promising genomic results and exemplify the potential of expanded studies examining single-nucleotide variants from CTCs, primary tumors, and metastases to better elucidate clonal disease progression in bladder cancer.

An ongoing study in a prospective multicenter clinical trial setting is in SWOG S1314, a randomized phase II testing a gene panel derived by co-expression extrapolation (COXEN)—as a predictive biomarker of response to neoadjuvant chemotherapy for localized, muscle-invasive bladder cancer. The primary aim of this study is to determine whether the COXEN score derived from CTC RNA can predict treatment response to neoadjuvant chemotherapy in a manner similar to RNA from bladder tumor biopsy. Collectively, these studies in locally advanced and metastatic bladder cancer aim to leverage improved recovery and amplification techniques to ultimately predict outcome and improve therapy selection.

### Kidney and Testis

Few studies have been conducted evaluating CTCs in either kidney (64–66) or testis cancer (67), and most have focused on CTC enumeration. Nel et al. (64) studied CTCs in metastatic renal cell carcinoma patients using multiparameter immunofluorescence microscopy and detected subtypes of CTCs consistent with epithelial, mesenchymal, and stem cell-like characteristics. Bluemke et al. (65) detected CTCs in 81 (53%) of 154 patients with renal cell carcinoma using autoMACS^®^, an immunomagnetic-based platform. They reported that CTC detection was associated with positive lymph nodes (*p* < 0.001), synchronous metastases (*p* = 0.014), and in multivariate Cox regression analysis, CTCs were associated with overall survival (RR, 2.3, *p* = 0.048). An interesting study from Nastaly et al. (67) evaluated CTC enrichment from peripheral blood as well as testicular vein blood in patients with testicular germ cell tumors (GCTs). CTC detection was associated with clinical stage, non-seminomatous GCTs, and elevated serum levels of α-fetoprotein, human chorionic gonadotropic, and lactate dehydrogenase. Furthermore, 14/122 (11.5%) of patients had detectable CTCs in peripheral blood, while 12/19 (63%) of patients had detectable CTCs in testicular vein blood. While these preliminary findings are promising, additional larger trials are needed in these tumor types to better define the future role of CTC analysis in these cancers.

## Summary and Future Directions

Whereas the majority of early CTC studies in GU cancer were focused on identification and enumeration—an end point with clinically important prognostic and predictive value—a growing emphasis now is being placed on molecular characterization of the tumor cells. As identification, recovery, and characterization become more accurate and cost effective, it will be important to determine whether the molecular profiles of CTCs provide information that can be used as a surrogate for tumor tissue, or whether in some cases CTCs provide information exceeding that available from biopsies (e.g., reflecting new drivers of resistance and progression). Gene rearrangements, translocations, differences in receptor expression and localization, and splice variations have all been successfully assayed in CTCs, and the molecular profiles derived from these liquid biopsies ultimately will offer utility in directing targeted therapy and selecting patients for appropriate clinical trials.

Future CTC studies in GU and other malignancies also will need to be integrated with rapidly emerging technologies for isolating and analyzing cell-free circulating tumor DNA (ctDNA), which is released from dying cells in primary tumors, metastases, and CTCs. Collection, shipping, and isolation of ctDNA are relatively straightforward and qPCR or NGS can be used to monitor cancer progression or emergence of new driver mutations (68). For example, through the use of ctDNA sequencing technology in the setting of CRPC, Lallous et al. (69) identified four single AR mutations and five mutation combinations associated with CRPC, which could be relevant to prognosis and therapy. In non-GU malignancies, ctDNA has demonstrated capacity as an accurate biomarker for extent of surgical resection and disease relapse (70) as well as a means of detecting therapeutically relevant DNA mutations in both the pre- and postsurgical settings (71). Just as CTC fragments were found predictive of prostate cancer recurrence, ctDNA offers a useful biomarker of residual disease or early disease recurrence and continues to be explored clinically with evolving applications (19, 72). An ongoing clinical trial, NCT02771769, is examining the utility of ctDNA in men with elevated PSA undergoing prostate biopsy to determine if copy number instability correlates with prostate cancer diagnosis and may reshape the landscape of prostate cancer screening (http://clinicaltrials.gov). As with CTCs, ctDNA analysis presents important new questions: for example, ctDNA represents the genome of dying tumor cells (as well as any other dying non-tumor cells), so the genomes of treatment-resistant cells may not be fully represented or detectable. Moreover, whereas CTCs allow multiparametric analysis of DNA, RNA, and proteins, ctDNA by definition yields just genomic information. Our own work in this field (unpublished data) suggests that—not unexpectedly—CTC-derived and cell-free-derived DNA may yield somatic profiles that do not fully overlap and may ultimately complement each other as liquid biopsy methods. Still other blood-based markers now emerging include tumor cell-derived exosomes (73) and epigenetic profiling of ctDNA (74, 75). The future of “liquid biopsy” therefore likely involves using all of these methods and better defining their respective utility in clinical settings (76).

## Author Contributions

CH and DZ assisted in researching, writing manuscript, and constructing associated figures. AG supervised work and assisted in research, writing, editing, and formatting of manuscript.

## Conflict of Interest Statement

The authors declare that the research was conducted in the absence of any commercial or financial relationships that could be construed as a potential conflict of interest.
